# Early allelopathic input and later nutrient addition mediated by litter decomposition of invasive *Solidago canadensis* affect native plant and facilitate its invasion

**DOI:** 10.3389/fpls.2024.1503203

**Published:** 2024-12-19

**Authors:** Jianfan Sun, Yundi Fu, Wenjie Hu, Yanwen Bo, Mohsin Nawaz, Qaiser Javed, Wajid Ali Khattak, Rasheed Akbar, Wang Xiaoyan, Wei Liu, Daolin Du

**Affiliations:** ^1^ Institute of Environment and Ecology, School of the Environment and Safety Engineering, Jiangsu University, Zhenjiang, China; ^2^ Jiangsu Collaborative Innovation Center of Technology and Material of Water Treatment, Suzhou University of Science and Technology, Suzhou, China; ^3^ Department of Agriculture and Nutrition, Institute of Agriculture and Tourism, Poreč, Poreč, Croatia; ^4^ Department of Entomology, Faculty of Physical and Applied Sciences, The University of Haripur, Haripur, Khyber Pakhtunkhwa, Pakistan; ^5^ College of Optical, Mechanical and Electrical Engineering, Zhejiang A&F University, Hangzhou, China; ^6^ Jingjiang College, Institute of Enviroment and Ecology, School of Emergency Management, School of Environment and Safety Engineering, Jiangsu University, Zhenjiang, China

**Keywords:** allelopathy, invasive species, carbon and nitrogen cycle, plant-soil-feedback, invasion success

## Abstract

Litter decomposition is essential for nutrient and chemical cycling in terrestrial ecosystems. Previous research on *in situ* litter decomposition has often underestimated its impact on soil nutrient dynamics and allelopathy. To address this gap, we conducted a comprehensive study involving both field and greenhouse experiments to examine the decomposition and allelopathic effects of the invasive *Solidago canadensis* L. in comparison with the native *Phalaris arundinacea* L. In the field, a 6-month litter bag experiment using leaf litter from *S. canadensis* and *P. arundinacea* was conducted across three community types: invasive, native, and mixed. Seed germination tests were also performed to investigate the allelopathic effects of decomposing litter. In the greenhouse, a pot experiment with lettuce as a bioindicator was performed to examine the allelochemical inputs from litter decomposition over various time intervals (0, 30, 60, 120, and 180 days). Subsequently, a soil–plant feedback experiment was carried out to further evaluate the effects of decomposing litter on soil biochemistry and plant dynamics. The findings of this study revealed that *S. canadensis* litter decomposed more rapidly and exhibited greater nitrogen (N) remaining mass compared with *P. arundinacea* in both single and mixed communities. After 180 days, the values for litter mass remaining for *S. canadensis* and *P. arundinacea* were 36% and 43%, respectively, when grown separately and were 32% and 44%, respectively, in mixed communities. At the invasive site, the soil ammonia and nitrate for *S. canadensis* increased gradually, reaching 0.89 and 14.93 mg/kg by day 120, compared with the native site with *P. arundinacea*. The soil organic carbon for *S. canadensis* at the invasive site also increased from 10.6 mg/kg on day 0 to 15.82 mg/kg on day 120, showing a higher increase than that at the native site with *P. arundinacea*. During the initial decomposition stages, all litters released almost all of their allelochemicals. However, at the later stages, litters continued to input nutrients into the soil, but had no significant impact on the soil carbon (C) and N cycling. Notably, litter-mediated plant–soil feedback facilitated the invasion of *S. canadensis*. In conclusion, this study highlights the significance of litter decomposition as a driver of transforming soil biochemistry, influencing the success of invasive *S. canadensis*.

## 1 Introduction

The invasion rate of exotic species is indeed expected to increase rapidly in the coming decades due to several key factors associated with human activities, such as travel and tourism, urbanization, and infrastructure development (Javed et al., 2023; Liao et al., 2008; Pyšek et al., 2020). Plant invasion is not only threatening to the biodiversity and stability of natives but also has profound impacts on ecosystem stability, trait composition, and ecosystem functioning and processes (Chase et al., 2020; Isbell et al., 2017; Pan et al., 2023). The introduction of an exotic plant species could alter the species composition by changing the richness and relative abundance of the native species, altering the carbon (C) and nitrogen (N) cycle (Yang et al., 2020; Zhang et al., 2019a), the soil N mineralization (van der Putten et al., 2013), the plant nutrient uptake (Zhang et al., 2019b), and the nutrient transfer to soil through litterfall (Zhang et al., 2018b; Zhu et al., 2022a). These changes could affect the different controls of the litter decomposition rates (Delgado-Baquerizo et al., 2015; Veen et al., 2015). In general, successful invaders often pose more and higher quality litter (e.g., a higher N content or a lower C/N ratio), which usually decomposes faster compared with native plants (Incerti et al., 2018; Zeng et al., 2020; Zhang et al., 2014). Invasive species, which are non-native plants or animals that invade new areas, often make litter (dead leaves and plants) decompose faster (Ullah et al., 2024; Zhang et al., 2016; Zhong et al., 2022). This happens because many successful invasive species are quick to spread and grow, and they do not put much effort into building permanent structures (Adomako et al., 2019; van Kleunen et al., 2010). This faster litter decomposition can affect the environment. Moreover, the rate of decomposition depends on the climatic variables, litter quality, soil biota, and decomposition time (Marchante et al., 2019). Many studies have looked at how leaves decompose and release nutrients, considering different plant species (Asplund and Wardle, 2017; Chomel et al., 2016; Fujii et al., 2017; Keller and Phillips, 2019). However, when it comes to invasive plants, researchers and scientists are still figuring out how litter decomposition affects the native biodiversity in different invasion extents and over different periods.

Litter decomposition plays a pivotal role within ecosystems, serving as a fundamental process that exerts control over nutrient cycling and energy flux (Findlay, 2021; Lambers et al., 2019). This, in turn, influences various ecological factors, including the composition of the soil organic matter, the availability of nutrients, and the structure and functions of communities (Bradford et al., 2016; García-Palacios et al., 2016a; García-Palacios et al., 2016b; Incerti et al., 2018). The process can be led by both biotic and abiotic factors (Petit-Aldana et al., 2019; Yue et al., 2018). The quality of leaf litter, particularly its nitrogen (N) concentration, plays a critical role in decomposition processes, primarily influencing the activity and composition of decomposer communities such as bacteria and fungi, subsequently affecting soil processes through feedback mechanisms. Many secondary metabolites are delivered into the soil during decomposition (Yan et al., 2018). These secondary metabolites have been considered as potential allelochemicals, exhibiting the capacity to endure in the soil post-senescence and to exert inhibitory effects on the growth and germination of neighboring and subsequent-generation plants (Bonanomi et al., 2011; Chomel et al., 2016; Del Fabbro and Prati, 2015; Zhang et al., 2019c). During litter decomposition, a large amount of water-soluble allelochemicals, such as total phenols and flavonoids, enters the soil quickly through rainfall patterns and changes the soil properties. The changes in the soil physiochemical properties induced by allelochemicals give positive feedback to invasive species over native species (plant–soil feedback) (Hättenschwiler and Vitousek, 2000; Weston and Mathesius, 2013). These chemical compounds provide more resources to decomposers and suppress the growth of native plants, consequently altering the diversity of native plant communities (Zhang et al., 2019d). Therefore, there is a need to understand at which stage or at which period these allelochemicals can trigger maximum plant invasion.

In addition, litter decomposition influences primary ecosystem productivity and greatly impacts ecosystem processes throughout invaded communities (Luo et al., 2018). The C and N cycles are indeed fundamental components of ecosystem functioning, driving essential processes that sustain life on Earth and play a vital part in the nutrient budget of an ecosystem (Zhang et al., 2020e). However, the C and N cycles could potentially influence the invasion process of alien plants, mainly through alterations in allelopathy (Raheem et al., 2024). These changes in their allelopathy affect the seed germination and seedling growth of adjacent species (Wang et al., 2020). Invasive species grow rapidly and accumulate more nitrogen in their tissues, which is subsequently returned to the soil upon decomposition in greater amounts than in indigenous plants, potentially leading to an increased availability of N at the soil surface (Adomako et al., 2021; Yelenik and D’Antonio, 2013). Therefore, the nutrient cycling rate in invaded areas could increase, and at the later stage of litter decomposition, the nutrient inputs might increase the invasion ability of invasive species (Zeng et al., 2020). Many studies have focused on the effects of litter decomposition, specifically on the C and N cycles or the secondary metabolite effects of isolated decomposition (such as allelopathy) (Chomel et al., 2016; Wolfe and Ballhorn, 2020; Zhang et al., 2019c). There are studies on the relationship between the C and N cycles and allelopathic effects, particularly on the coexisting effects of cyclic and secondary metabolites (Macías et al., 2019; Scavo et al., 2018). Recent studies (Macías et al., 2019; Scavo et al., 2018) have explored the influence of the litter decomposition of invasive species relative to that of native species, investigating the associated mechanisms (Dignac et al., 2017; Finch et al., 2021). However, the allelopathic effects of litters derived from invasive, native, and mixed cultures under different time frames have not been studied yet in terrestrial ecosystems.

In this study, we focused on how litter decomposition from invasive and native plants affects the soil biochemistry and nutrient cycles over time, using both field and greenhouse experiments. This approach allowed assessing the impacts on the C and N cycles, allelochemicals, and plant–soil feedback mechanisms. This study aimed to deepen our understanding of plant–soil interactions and the mechanisms driving ecosystem processes. It was hypothesized that a rapid release of allelochemicals in the initial stages of litter decomposition, which might disrupt the soil chemistry, followed by a steady input of nutrients in the later stages, could potentially enhance the invasion rates and limit native plant growth. This overall process—known as plant–soil feedback—could ultimately support invasive species while negatively affecting native plants. Accordingly, the study objectives were as follows: 1) to evaluate the effect of the litter decomposition rates on soil conditions and invasive success relative to native species; 2) to characterize the decomposition dynamics across multiple time intervals; and 3) to investigate the plant–soil feedback effects of invasive litter and their impact on local plant communities.

## 2 Materials and methods

### 2.1 Study site

The study was conducted in Jiaoshan, Zhenjiang City, Jiangsu Province, China (32°9′ N, 119°31′ E), in 2018. The annual average temperature was 15.6°C, while the monthly average was from −10°C (January) to 31.3°C (July). The annual rainfall was 1,088.2 mm. The soil, characterized by its high clay content, is classified as Gleysol or water-soluble soil. The selected study site was dominated by native *Phalaris arundinacea* L., *Phragmites australis* (Cav.) Trin. ex Steud., *Zizania caduciflora* Hand.-Mazz., *Polygonum lapathifolium* L., and *Cardamine lyrata* Bunge and was invaded by *Solidago canadensis*. The native *Phalaris* spp. covered approximately 60% of the area, while *S. canadensis* accounted for approximately 20%.

### 2.2 Experiment 1: Litterbag experiment

**Figure 1:**
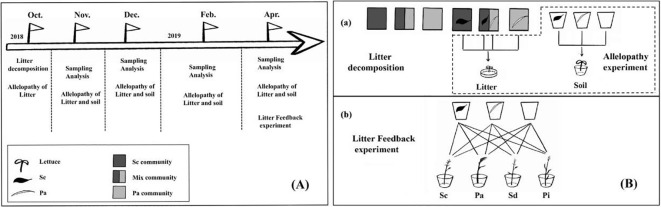
Experimental design. **(A)** The litter decomposition experiment was conducted from October 2018 to April 2019. The experiment was divided into *in situ* decomposition experiments and greenhouse supplement experiments. Three plant community plots for the *in situ* decomposition experiments were selected based on the degree of *Solidago canadensis* invasion. **(B)** Litter and soil samples were recovered for analysis in months 1, 2, 3, 4, and 6. At the same time, the allelopathic effect of the recovered litter extract was examined. In order to investigate the metabolic dynamics of litter allelochemicals entering the soil, litter decomposition was also carried out in the greenhouse, and litter was taken out at months 1, 2, 3, 4, and 6; lettuce was grown using the soil. In addition, after decomposing the litter in the greenhouse for 6 months, *Solidago canadensis* (*Sc*), *Phalaris arundinacea* (*Pa*), *Solidago decurrens* (*Sd*), and *Pterocypsela laciniata* (*Pl*) were planted to assess the soil feedback of the decomposition of *Sc* litter.

The plant communities selected were 1) highly invaded communities (invaded by invasive *S. canadensis*); 2) mixed communities (a 50:50 ratio of *S. canadensis* to *P. arundinacea*); and 3) native *P. arundinacea* communities without *S. canadensis* invasion. Each community was replicated three times for nine sites (three sites per community) (**Figure 1**).

All sites were placed within 1 km and were chosen to have similar characteristics, such as slope, temperature, and light, among others. Dynamic changes in the litter decomposition of both native and invasive species were studied through *in situ* litterbag experiments. In September 2018, wholly developed mature leaf litter samples from different *S. canadensis* and *P. arundinacea* individuals in the invaded area were collected manually. Air-dried litter samples were cut into sizes of 3–6 cm (approximately 5 g) of a single species and placed in nylon litterbags (1 mm mesh, 10 × 15 cm) (Lecerf, 2017). All woody and non-woody litters were carefully removed in each plot, and live or dead plant material was removed. The two litters (woody and non-woody) were weighed separately, equal proportions of each litter type were removed by treatment level, and then the remaining litters were returned to the plots in October 2018. A total of 10 replicates of each treatment were obtained after 30, 60, 120, and 180 days of litter decomposition; the others were analyzed at 0 month. Once the bags were removed, the litter samples were processed, sorted by species, dried, and weighed. The C and N in the litter types after different periods were measured using an elemental analyzer. Determination of the cellulose and lignin contents in the litter was performed using sulfuric acid extraction of anthrone (Gessner, 2005).

### 2.3 Experiment 2: Litter decomposition and allelopathy experiment

A seed germination experiment was performed to analyze the litter allelopathic effect. The allelopathic effect (RIs) indices were determined to evaluate the allelopathic effects of the litter extracts on lettuce seed germination and seedling growth (An, 2005; Wang et al., 2019). Lettuce seeds are highly sensitive to allelochemicals, and they are often used in allelopathic research experiments (Thiébaut et al., 2019; Yuan et al., 2013); hence, lettuce was selected as the test plant. The retrieved littered leaves at each fabricated littered time were placed into a flask filled with 100 ml of distilled water, soaked at room temperature for 48 h, and then filtered. The derived solution was then placed in a refrigerator at 4°C for subsequent processing. Five replicates were set for each treatment using distilled water as a control. To reduce the sensitivity difference of lettuce seeds to allelochemicals caused by storage time, a control experiment with distilled water was carried out at each decomposition stage. Seed germination and growth tests were carried out using culture dishes.

The surface of the lettuce seeds was sterilized with 1% sodium hypochlorite for approximately 15 min and then rinsed thoroughly with deionized water. Two layers of filter paper were spread on the bottom of a Petri dish (diameter, 9 cm), and 30 healthy lettuce seeds were then carefully moved into the Petri dish. Subsequently, 5 ml of the aforementioned treatment solution was added to each. The culture dishes were stored in a temperature-controlled incubator at 25°C for 10 days, with the temperature set at 25°C for 8 h, 20°C for 8 h, and 16°C for 8 h. Leaf litter extract or deionized water of approximately 0.5 ml was added daily to each Petri dish. The number of seeds that were germinated was counted daily after sprouting. After 14 days of cultivation, 10 seedlings were randomly selected from each Petri dish to measure the lettuce seed germination and seedling growth index values. The total phenol and flavonoid contents were determined using the Folin–Ciocalteu colorimetric method and ultraviolet spectrophotometry (Bärlocher and Graça, 2005), respectively.

At the same time, the dynamic effects of allelochemicals in the litter types were investigated through greenhouse pot experiments. The leaf litter samples from *S. canadensis* and *P. arundinacea* were buried in the flower pots (5 g per 1 kg of soil) taken 30, 60, 120, and 180 days after decomposition. A blank soil without added litter was also considered a control. Five replicates were set for each treatment. The soil with added litter was mixed evenly and the lettuce plants grown accordingly. The plant growth parameters were measured after 1 month of sowing.

Soil samples were taken and mixed. An aliquot of five soils per site was stored at a field moisture content of 4°C to analyze the activities of ammonia, nitrate nitrogen, and others. Another aliquot of soil was air dried at room temperature, crushed to pass through a 2-mm sieve to remove large fragments of organic matter, and stored in a lined paper bag for chemical analysis, including total organic carbon (TOC), catalase, etc. The potassium dichromate volumetric method was used to measure the TOC (Parsons, 1962), and the soil catalase activity was determined using the 0.1 N KMnO_4_ titration method—determination of soil polyphenol oxidase activity by pyrogallol colorimetry. The urease activity was measured using a spectrophotometer at 578 nm/g dry soil in 24 hands, while the 3,5-dinitrosalicylic acid technique was used to measure the invertase activity. Data were read with a spectrophotometer at 508 nm and expressed as micrograms of glucose produced per gram of dry soil in 24 h (Burns, 1982; Marx et al., 2001; Zhou and Zhang, 2014).

### 2.4 Experiment 3: Litter feedback experiment

Additional experiments were performed to examine the soil feedback effects of the litter decomposition of *S. canadensis* and *P. arundinacea*. Standard two-phase experiments consisting of conditioning and feedback phases were conducted (Pernilla Brinkman et al., 2010). For the conditioning phase, in the greenhouse, the litter of each species was decomposed in pots (height, 16.5 cm; top diameter, 32 cm) filled with 4:1 organic nutritive soil and litter obtained from the *in situ* experiment. In each pot, 4 kg of soil was placed, and litter was added in proportion to 5 g of litter in 1 kg of soil. An empty litter bag was set as a control, and each group was repeated five times (Reinhart and Rinella, 2016). These 15 pots corresponded to the conditioning duration of 6-month litter decomposition periods. All of the litter was carefully removed from the soil at the end of the conditioning phase of the experiment in April 2019.

In the feedback phase, each soil mixture (from the previous experiment) was filled into 20 pots measuring 16 cm × 16 cm × 16 cm. *S. canadensis* and *P. arundinacea* were selected for the feedback phase. At the same time, *Pterocypsela laciniata* and *Solidago decurrens* were planted to examine the effects of feedback on other native plants. The plants were grown for 9 weeks in the experimental garden. All plants were harvested at the end of the feedback experiment in June 2019. Five mature and fully expanded leaves per individual plant were randomly selected to estimate plant growth performance. Subsequently, the aboveground and separated parts were segregated, ground to a constant weight, and weighed.

### 2.5 Statistical analysis

Two-way analysis of variance (ANOVA) was used to measure the differences between the plant growth performance and the soil physicochemical properties and between all treatment groups. The Student–Newman–Keuls test was followed for multiple comparisons, with significant differences at *p* ≤ 0.05. All statistical analysis was performed on the IBM SPSS Statistics software (version 25.0; IBM Corp., Armonk, NY, USA). ANOVA was also used to measure the effects of mixtures, incubation time (days), and their interactions with the litter mass loss, allelopathy, and soil physicochemical properties. Redundancy analysis (RDA) was used to measure the relationship between the soil biochemical characteristics and C substrate utilization. This method was designated according to an early detrended correspondence analysis with a gradient length <3.0, suggesting that a linear ordination model was used. The significance of the soil biochemical variables in explaining the variance in C substrate utilization was assessed using Monte Carlo simulations. The RDA was performed using CANOCO 4.5, and outcomes were visualized with the extension CanoDraw for Windows.

## 3 Results

### 3.1 Litter mass remaining

**Figure 2:**
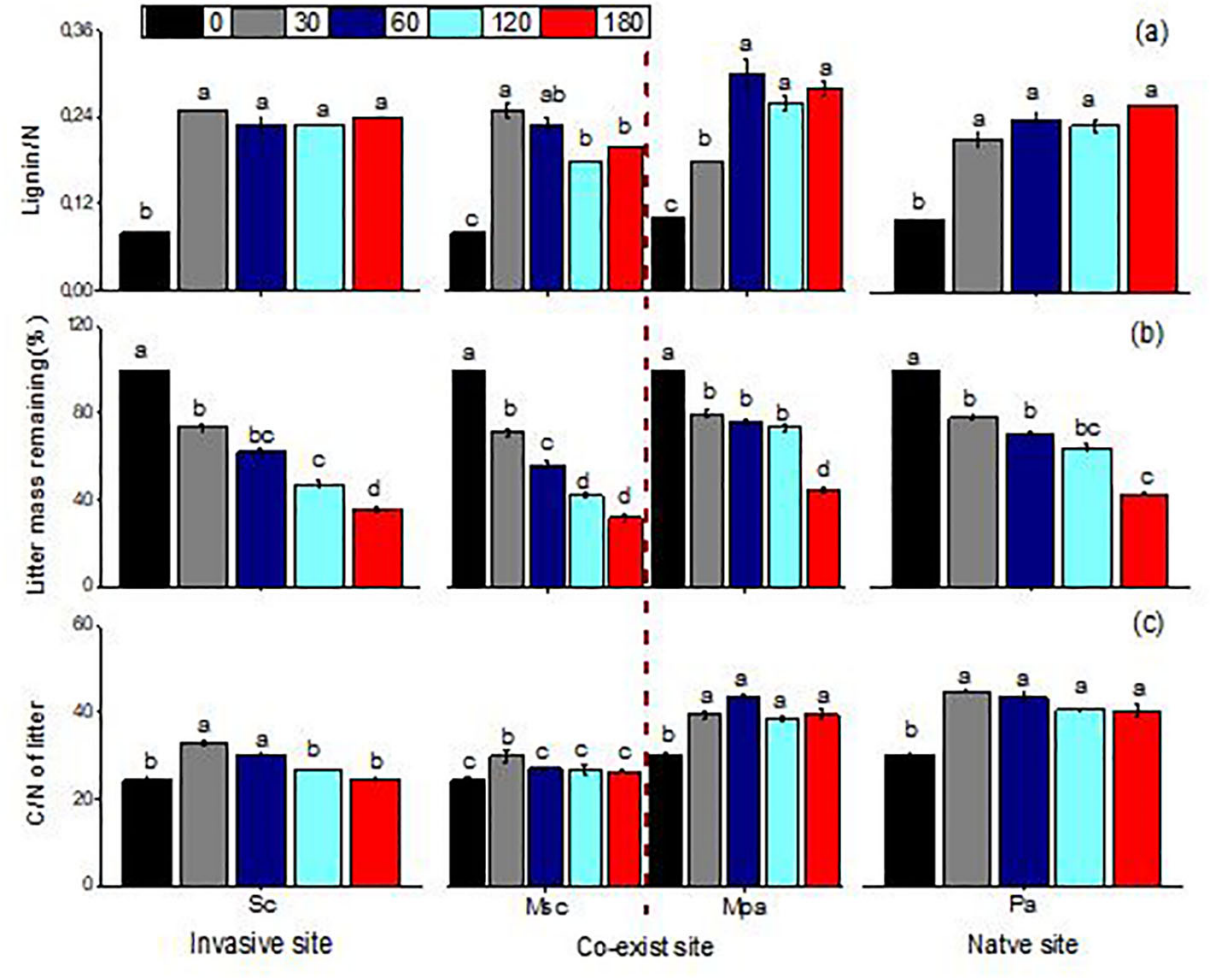
Lignin/nitrogen ratio **(A)**, litter mass remaining **(B)**, and carbon/nitrogen ratio of litter **(C)** (mean ± SE) of *Solidago canadensis* and *Phragmites* when each was decomposed alone or in mixed communities. *Sc*, *Solidago canadensis* at the invasive site; *Pa*, *Phalaris arundinacea* at the native site; *MSc*, *S. canadensis* in the mixed community at the co-exit site; *MPa*, *P. arundinacea* in the mixed community at the co-exit site.

Litter mass remaining (LMR), the C/N ratio, and lignin/N varied significantly across different litter types (i.e., invasive, native, and mixed), sampling dates, and their interactions (**Table 1**). When decomposed separately, *S. canadensis* litter degraded faster than *P. arundinacea* litter. In mixed communities, however, *S. canadensis* showed a slower decomposition rate, while *P. arundinacea* decomposed more rapidly. After 180 days, the LMR values for *S. canadensis* and *P. arundinacea* were 36% and 43%, respectively, in single-species settings compared with 32% and 44%, respectively, in mixed communities (**Figure 2**). In all three litter types, the C/N ratio gradually decreased as decomposition progressed (**Figure 2**). The lignin/N ratio in *P. arundinacea* litter increased until day 60 and then stabilized at this level for the rest of the study period. Conversely, in *S. canadensis* litter, the lignin/N ratio increased only during the initial stages at the invasive site, but showed a steady decline in the mixed or co-existing communities (**Figure 2**).

**Table 1:** Summary of the results of the two-way analysis of variance (ANOVA) of the physical properties of litter and the allelopathic effect index of the litter extracts.

Parameter	Factor	*Solidago canadensis* alone			*Solidago canadensis* mixed		
		*df*	*F*	*p*	*df*	*F*	*p*
Mass loss	Species	1	133.699	**<0.001**	1	565.735	**<0.001**
	Time	2.226	584.297	**<0.001**	2.917	573.347	**<0.001**
	Species*Time	2.226	10.483	**<0.001**	2.917	38.152	**<0.001**
Leaf C/N	Species	1	414.790	**<0.001**	1	2,287.776	**<0.001**
	Time	4	98.327	**<0.001**	4	26.978	**<0.001**
	Species*Time	4	18.189	**<0.001**	4	10.910	**<0.001**
Lignin/N	Species	1	9.005	**0.04**	1	109.438	**<0.001**
	Time	2.14	345.396	**<0.001**	4	157.277	**<0.001**
	Species*Time	2.14	11.848	**0.003**	4	41.966	**<0.001**
Total phenol	Species	1	15.685	**0.004**	1	5.921	**0.041**
	Time	1.062	158.926	**<0.001**	1.055	174.160	**<0.001**
	Species*Time	1.062	64.989	**<0.001**	1.055	73.709	**<0.001**
Total flavonoid	Species	1	266.029	**<0.001**	1	288.309	**<0.001**
	Time	1.06	487.424	**<0.001**	1.053	495.218	**<0.001**
	Species*Time	1.06	352.951	**<0.001**	1.053	350.899	**<0.001**
Carbon loss	Species	1	440.842	**<0.001**	1	1,6432.781	**<0.001**
	Time	4	1,007.088	**<0.001**	4	1,399.460	**<0.001**
	Species*Time	4	21.242	**<0.001**	4	119.502	**<0.001**
Nitrogen loss	Species	1	3.628	0.13	1	769.617	**<0.001**
	Time	4	1,520.748	**<0.001**	4	718.338	**<0.001**
	Species*Time	4	4.973	**0.008**	4	17.850	**<0.001**

*F* values and their significance are provided, and *p*-values <0.05 are highlighted in bold.

### 3.2 Allelopathy and nutrient input

**Figure 3:**
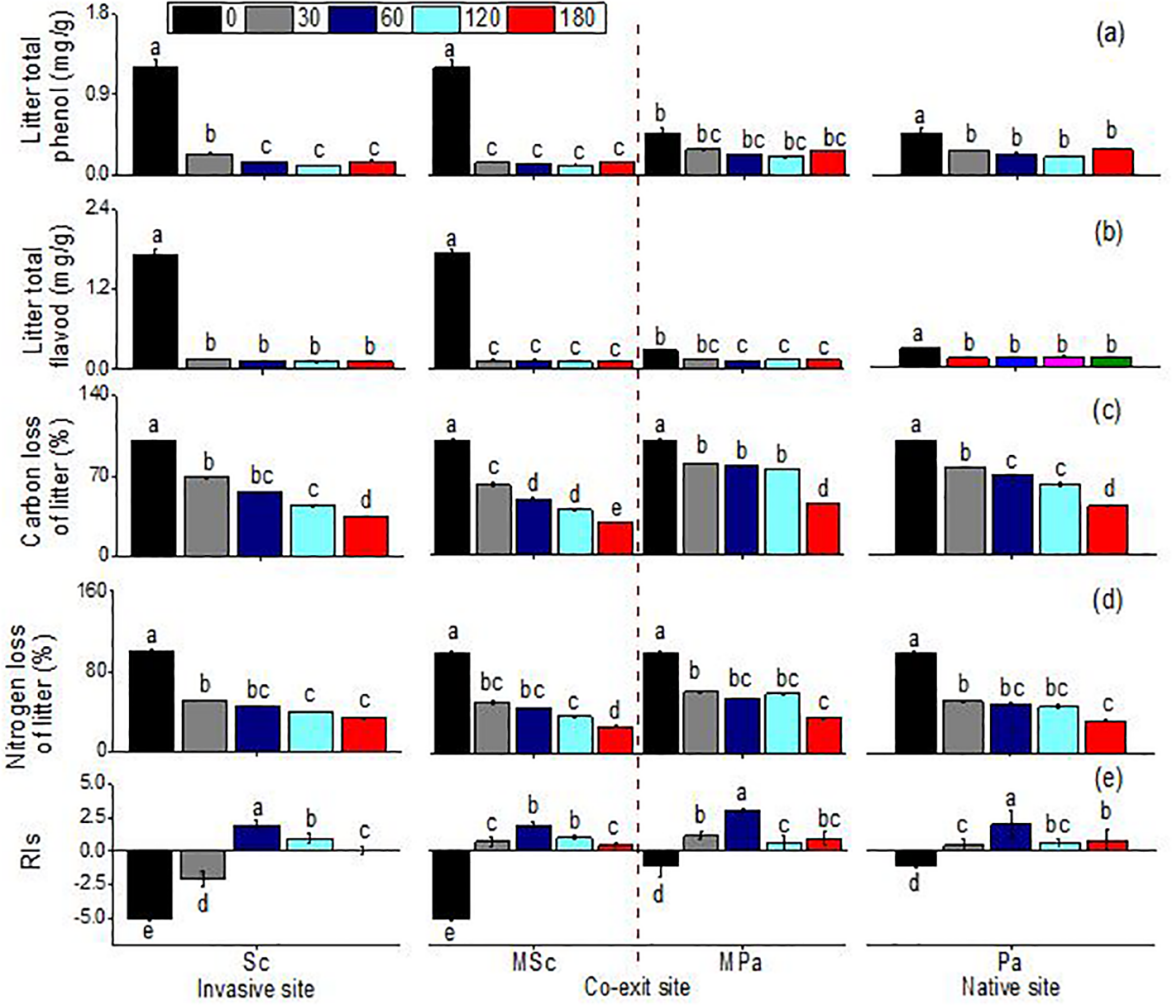
Total phenol **(A)**, total flavonoids **(B)**, carbon loss **(C)**, nitrogen loss **(D)**, and indices of allelopathic effects (RIs) **(E)**, (mean ± SE) of litter when each was decomposed alone or in mixed communities. *Sc, S. canadensis* at the invasive site; *Pa, P. arundinacea* at the native site; *MSc, S. canadensis* in mixed community at co-exit site; *MPa, P. arundinacea* in mixed community at co-exit site.

Generally, leaf extracts significantly affect the seedling growth indices of lettuce. The total phenol content, the total flavonoid content, and the carbon and nitrogen mass remaining significantly changed across different litter types (i.e., invasive, native, and mixed), sampling dates, and their interactions (**Table 1**). Leaf extracts, regardless of species, significantly decreased the RIs. Before decomposition, compared with that of *P. arundinacea*, the allelopathic effect of *S. canadensis* was more negative. After 30 days of decomposition, the allelopathic effects of the extracts of the two species were significantly weakened, and there was almost no allelopathic effect. Only the *S. canadensis* litter in single communities had a negative effect on the lettuce seeds. After 60 days of decomposition, this trend was reversed, and the extracts of the *S. canadensis* and *P. arundinacea* litters promoted the growth of lettuce, with the *P. arundinacea* litter in the mixed communities having the most significant growth promotion. Thereafter, the indices of the allelopathic effects slowly decreased and remained constant (**Figure 3**). There were some differences in the total phenol and total flavonoid contents between the two species, with the *S. canadensis* litter showing higher total phenol and total flavonoid contents; however, both species followed similar trends along time: In the first month, the total phenolic and total flavonoid contents decreased sharply and remained stable for 6 months. At the same time, the total phenol remaining in the *P. arundinacea* litter was higher than that in the *S. canadensis* litter in the next five 5 months (**Figure 3**). The LMR (carbon) of *S. canadensis* in the co-existing site was 100%. In contrast, the mass remaining (carbon) of the natives in the co-existing litter was 100% initially at 0 day; later, at 30, 60, and 120 days, the remaining carbon mass values of the natives in the coexisting sites were constant, 79.43%, 77.25%, and 74.82%, respectively (**Figure 3**). When the *S. canadensis* and *P. arundinacea* litters were decomposed in single communities, there were no differences in the rates of nitrogen loss. However, the mixed communities accelerated the litter nitrogen loss rate of *S. canadensis* in the co-exiting site (**Figure 3**).

**Figure 4:**
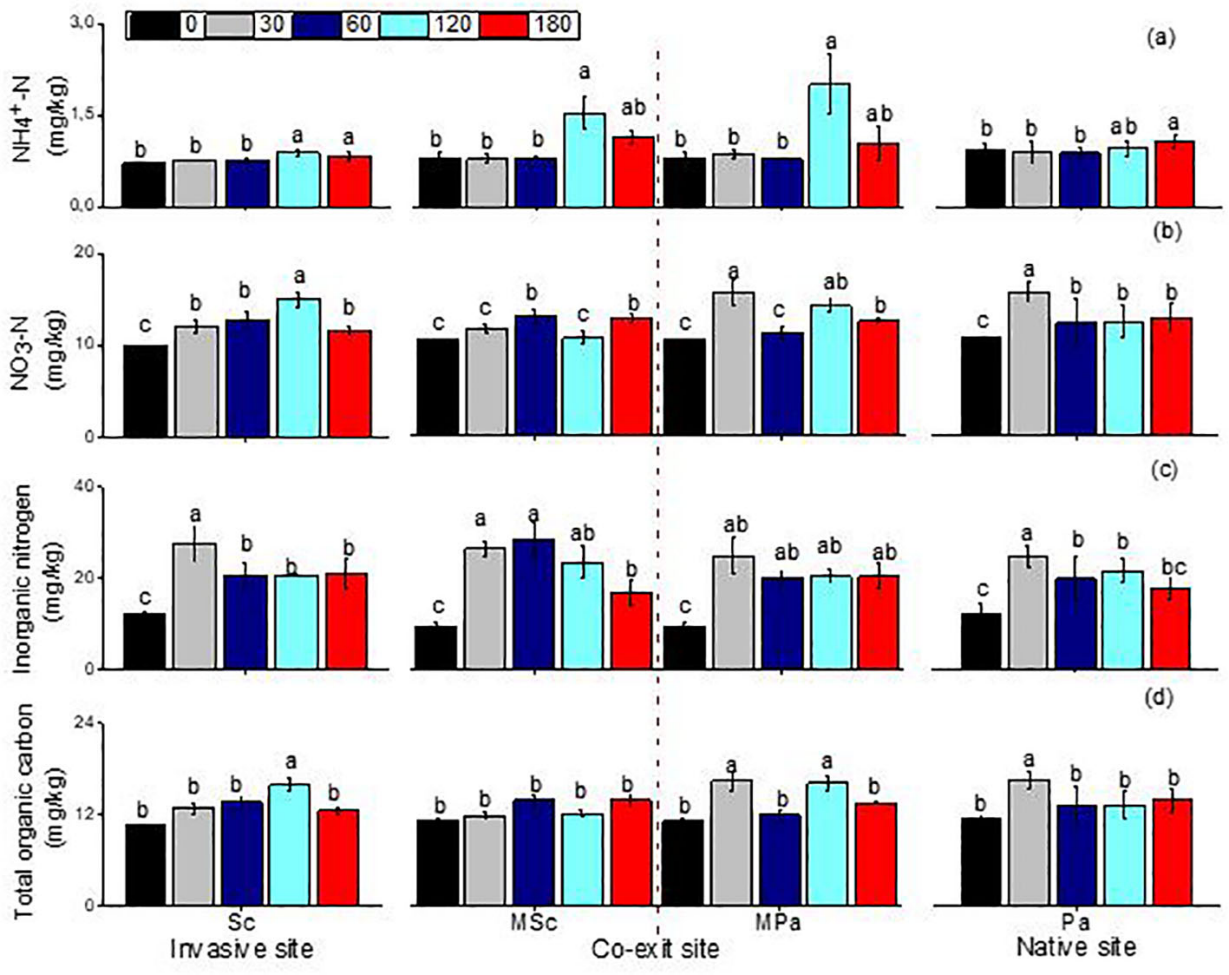
Linear fitting of ammonia nitrogen **(A)**, nitrate nitrogen **(B)**, soil inorganic nitrogen **(C)**, and total organic carbon **(D)** (mean ± SE) of soil when litter was decomposed alone or in mixed communities. *Sc*, *Solidago canadensis* at the invasive site; *Pa*, *Phalaris arundinacea* at the native site; *MSc*, *S. canadensis* in the mixed community at the co-exit site; *MPa*, *P. arundinacea* in the mixed community at the co-exit site.

The ammonia and nitrate, organic carbon, and inorganic nitrogen were significantly impacted by the different litter types (i.e., invasive, native, and mixed), the sampling dates, and their interactions (**Table 2**). The ammonia and nitrate in *S. canadensis* at the invasive site increased gradually up to 120 days to 0.89 and 14.93 mg/kg, respectively, compared with those in *P. arundinacea* at the native site. The soil organic carbon in *S. canadensis* at the invasive site increased gradually from 0 day to 120 days, from 10.6 to 15.82 mg/kg compared with *P. arundinacea* at the native site (**Figure 4**). The inorganic nitrogen in *S. canadensis* at the invasive and co-existing sites increased from 0 day to 60 days and decreased slowly from 60 days to 180 days (**Figure 4**).

**Table 2:** Summary of the results of the two-way analysis of variance (ANOVA) for the soil physiochemical data.

Parameter	Factor	*Solidago canadensis* alone			*Solidago canadensis* mixed		
		*df*	*F*	*p*	*df*	*F*	*p*
Soil C	Species	3	120.367	**<0.001**	2	76.079	**<0.001**
	Time	4	21.945	**<0.001**	2.835	4.639	**<0.016**
	Species*Time	12	18.794	**<0.001**	5.671	3.627	**<0.018**
Soil N	Species	3	163.724	**<0.001**	2	884.74	**<0.001**
	Time	4	3.465	**<0.018**	4	2.817	**<0.048**
	Species*Time	12	26.645	**<0.001**	8	17.535	**<0.001**
TOC	Species	3	1.445	0.3	2	9.142	**0.015**
	Time	4	9.646	**<0.001**	4	20.096	**<0.001**
	Species*Time	12	0.672	0.764	8	4.130	**0.003**
Ammonium nitrogen	Species	3	4.784	**0.034**	2	2.285	0.183
	Time	4	3.482	**0.018**	1.184	5.473	**0.048**
	Species*Time	12	0.402	0.952	2.368	0.983	0.434
Nitrate	Species	3	6.001	**0.019**	2	22.335	**0.002**
	Time	1.49	3.887	0.06	4	5.336	**0.003**
	Species*Time	12	2.135	**0.043**	8	7.028	**<0.001**
Inorganic nitrogen	Species	3	5.783	**0.021**	2	24.459	**0.001**
	Time	1.522	4.073	0.053	4	5.632	**0.002**
	Species*Time	4.567	2.191	0.126	8	7.007	**<0.001**
Polyphenol oxidase	Species	3	4.962	**0.013**	2	0.824	0.462
	Time	1.705	667.063	**<0.001**	1.795	240.160	**<0.001**
	Species*Time	5.115	16.879	**<0.001**	3.59	5.469	**0.004**
Catalase	Species	3	3.609	**0.037**	2	3.046	0.085
	Time	2.236	38.355	**<0.001**	1.61	15.373	**<0.001**
	Species*Time	6.707	2.168	0.063	3.22	0.439	0.741
Urease	Species	3	4.754	**0.015**	2	24.074	**<0.001**
	Time	1.268	0.963	0.36	1.196	0.538	0.506
	Species*Time	3.803	0.593	0.664	2.392	1.148	0.354
Sucrase	Species	3	25.955	**<0.001**	2	36.602	**<0.001**
	Time	2.759	11.723	**<0.001**	4	3.832	**0.009**
	Species*Time	8.276	4.325	**0.001**	8	6.234	**<0.001**

*F* values and their significance are provided, and *p-*values <0.05 are highlighted in bold.

### 3.3 Soil enzyme activity

The soil enzyme activity, including polyphenol oxidase, catalase, and sucrase, exhibited significant variations based on the soil intrusion plots, decomposition duration, and their interactions (**Table 2**). Notably, after 60 days of litter decomposition, the polyphenol oxidase activity was significantly inhibited, while the catalase activity showed a marked increase on the same day. The sucrase activity remained stable at 30 and 180 days post-decomposition, but showed a notable increase during the mid-stages, specifically on days 60 and 120. In contrast, the urease activity did not exhibit significant changes in response to the decomposition time or its interaction with other factors (**Table 2**).

### 3.4 Soil feedback

**Figure 5:**
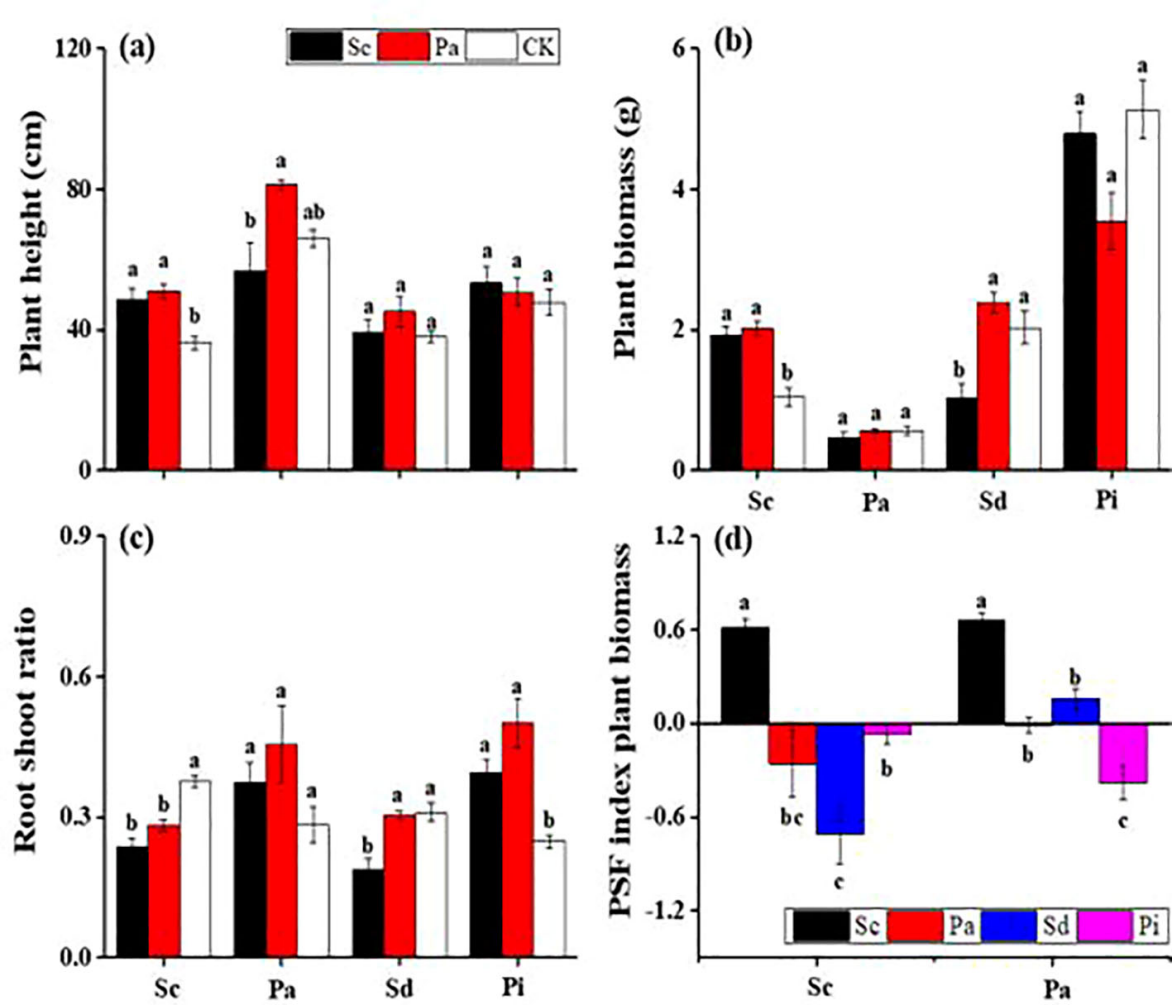
Plant height **(A)**, plant biomass **(B)**, root-to-shoot ratio **(C)**, and plant–soil feedback (PSF) index plant biomass **(D)** (mean ± SE) of *Solidago canadensis* (*Sc*), *Phalaris arundinacea* (*Pa*), *Solidago decurrens* (*Sd*), and *Pterocypsela laciniata* (*Pl*) when grown in different feedback soils.

In invaded soils, the growth of *P. arundinacea* plants was significantly suppressed, with a reduction of approximately 30% compared with those in native soils (**Figure 5**). The lowest recorded plant height was 39.2 cm, which was for *S. decurrens* in invaded soil conditions. In addition, the invaded soils negatively impacted both the root-to-shoot ratio and the biomass of *P. arundinacea* and *S. decurrens* plants (**Figure 5**). Analysis of the plant–soil feedback interactions revealed a consistent negative effect associated with soils conditioned by invasive *S. canadensis*, leading to diminished performance in native species such as *P. arundinacea*, *S. decurrens*, and *P. laciniata*. Conversely, invasive *S. canadensis* exhibited a positive feedback interaction and showed positive values in native and invaded soils (**Figure 5**).

### 3.5 Interaction between soil biochemical characteristics and allelochemical analysis

**Figure 6:**
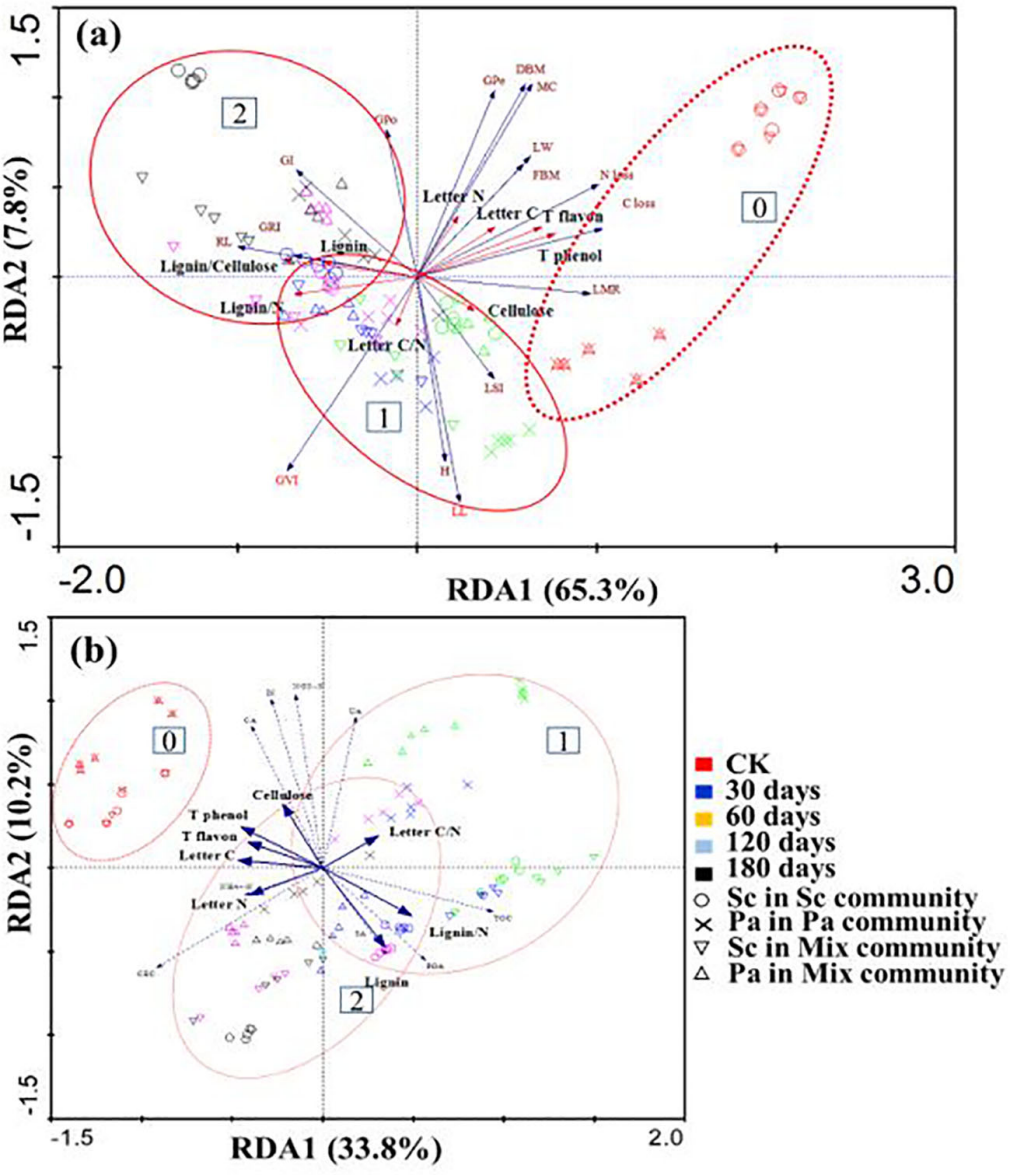
Result of redundancy analysis (RDA) **(A)** based on allelopathy, litter loss, soil nutrition, and impact factors (Cellulose, Lignin, Lignin/cellulose, Lignin/N, Quality loss, Total phenol, and Total flavon). Redundancy analysis (RDA) **(B)** based on litter decomposition days and plant communities. *C/N* is the litter carbon to nitrogen ratio, *NH_4_^+^–N* is Ammonium nitrogen, *NO_3_^-^–N* is Nitrate, *IN* is Inorganic nitrogen, *POA* is Polyphenol oxidase, *CA* is Catalase, *UA* is Urease, *SA* is Sucrase, *RL* is Root Length, *H* is Plant height, *LL* is Leaf length, *LW* is Leaves wide, *LSI* is Leaf index, *FBM* is Fresh weight of seedlings, *DBM* is Dry weight of seedlings, *MC* is Water content, *GFe* is Germination rate, *GPo* is Germination potential, *GI* is Germination index, *GVI* is Germination vitality index, *GRI* is Germination speed index. Data were grouped according to the decomposition time (0 months, 2 months after the initial decomposition, and late decomposition).

In the RDA of allelopathy, litter loss, and soil nutrition with an environmental factor as the explanatory variables, axis 1 (the *x*-axis) accounted for 65.3% and 33.8% of the variation in the dataset, with 7.8% and 10.2% of the variation accounted for by axis 2 (the *y*-axis). Together, 95.8% and 91.5% of the variation in allelopathy and LMR, respectively, were explained (**Figures 6A, B**). In general, the decomposition time contributed more to the separation of the samples. Allelochemicals (total phenols and total flavonoids) mainly negatively controlled the root length, C loss, N loss, and LMR, which were positively correlated with the litter C and N contents and negatively correlated with lignin/N and C/N (**Figure 6A**). At the same time, inorganic nitrogen and lignin/N were also negatively correlated (**Figure 6**).

## 4 Discussion

### 4.1 Litter quality and the soil matrix affect litter decomposition

In the present study, the *S. canadensis* and *P. arundinacea* litters in mixed communities resulted in non-additive effects on decomposition compared with the litter from each species decomposing in single communities. Our research showed that *S. canadensis* had a lower C/N ratio than *P. arundinacea*, and its mass remaining was higher and its nitrogen release faster than that of *P. arundinacea* (**Figure 2**). The results from the present study aligned with those of previous studies showing that litter characteristics such as high-quality litter (litter with a low C/N ratio) usually decompose faster than low-quality litter (Cornwell et al., 2008; García-Palacios et al., 2016; Wu et al., 2023). This might have occurred due to high-quality litter (low C/N ratio and low lignin) decomposing more rapidly in a well-structured, nutrient-rich soil, but slowly in nutrient-poor or compacted soils. However, the soil texture can influence the decomposition of different types of litter differently (Angst et al., 2021). For instance, fine-textured soils (high clay content) might slow down the decomposition of high-lignin litter more than coarse-textured soils.

On the contrary, low-quality litter (high lignin content) might decompose more efficiently in soils with a diverse and active microbial community capable of breaking down complex compounds (Kohmann et al., 2020; Pasricha, 2017). Therefore, the lignin/N ratio was also considered an extremely important litter chemical property for describing the litter quality in our study (Kuebbing et al., 2019). At the same time, the decomposition of litter and the release of carbon were hindered on the 60th day (**Figures 2**, **3**), which might be due to the polyphenol oxidase activity in the soil being inhibited, which could be related to the release of secondary metabolites in the litter. The additive effect could be due to *S. canadensis* changing the soil properties, while the secondary compound release was associated with its negative effects. This indicates that the soil properties have an additive effect on the litter decomposition process and the litter quality (Bani et al., 2018; Lu et al., 2017; Ristok et al., 2017; Yan et al., 2018). For example, the litter composition of *Spartina alterniflora* and *Phragmites communis* selects different soil microbial structures and controls the different litter decomposition patterns and soil carbon sequestration capacity (Yan et al., 2018). Overall, our results showed that not only the quality of litter but also the soil comprised the main driving force in the decomposition of litter. Understanding these interactions helps in the prediction of the decomposition rates and nutrient cycling in various ecosystems.

### 4.2 Release of allelochemicals

The leaves of *S. canadensis* and *P. arundinacea* can release allelochemicals such as catalase and phenols during the initial decomposition process. The allelopathic activity of these compounds inhibited lettuce seed germination and seedling growth, consistent with previous studies (Del Fabbro and Prati, 2015; Wang et al., 2019). At the same time, the *S. canadensis* litter contained higher levels of allelochemicals and showed faster decomposition, providing evidence of the good invasion of this species. The chemical composition of the litter and the difference in the allelopathic effects also indicated that the allelopathic effect of the litter was highly time-sensitive and that this effect may be reduced during the decomposition process. In addition, the timing of allelochemical release, particularly during early decomposition, maximizes their inhibitory effects on native plants when they are most vulnerable (e.g., germination and early growth stages) (Scavo et al., 2019a). As decomposition progresses, nutrient release could shift from allelopathic inhibition to nutrient enrichment (Xu et al., 2023), allowing *S. canadensis* to establish itself in the soil it has conditioned, further enabling its dominance. When plant litter is freshly deposited at the initial stage, it often contains high concentrations of allelochemicals (Santonja et al., 2019). These compounds can leach into the soil with the first rain or through the action of dew, which has a strong inhibitory effect on seed germination and the growth of neighboring plants (Einhellig, 2018; Macías et al., 2020). In the final stage of decomposition, the litter is largely broken down into basic nutrients and humus. By this stage, most of the original allelochemicals have either degraded or been assimilated by soil microorganisms (He et al., 2020). Soil allelopathic experiments have shown that allelochemicals inhibit the growth of lettuce seeds at the initial stage of growth (**Figure 5**). The effects of allelochemicals in the soil were more extensive, inhibiting germination and reducing the nitrogen and carbon rates (**Figure 3**). For example, the allelopathic compounds released by *S. canadensis* restrained the soil mineralization and nitrification processes at the high extract level (Zhang et al., 2014). Some chemicals are decomposed or transformed by microorganisms in the soil and can also provide abundant carbon sources for soil microbial growth, thus changing the function and structure of the microbial communities (Louvel et al., 2011; Qin et al., 2014; Zhu et al., 2017b). In a broader context (i.e., plant communities), allelochemicals can help explain the effects of litter mixture on the rate of litter decomposition and the adaptation/selection of soil organisms that lead to “homeland advantage.” However, this time-sensitive nature of the allelopathic effects of plant litter is influenced by various factors, including microbial activity, environmental conditions, and the chemical properties of the allelochemicals (Misra et al., 2020). Understanding these processes is essential for effectively managing allelopathy in agricultural and natural ecosystems.

### 4.3 Effects of litter decomposition on the soil C and N cycle

In the present study, although no significant changes were found, the variables associated with the C and N cycles in the soil tended to increase with litter decomposition, and increases in the litter input did not significantly affect the functions of the surface soil biota (**Figures 2**, **3**). The impact of litter decomposition on nutrient cycling might be minor in some cases, but it can significantly affect the biotic indicators, particularly native and invasive plant communities (Marchante et al., 2019; Souza-Alonso et al., 2024; Vilà et al., 2011). There are reasons for how litter decomposition impacts nutrient cycling and affects the native and invasive communities. Firstly, the response of ecosystem processes, such as the C and N cycles, to plant invasion can indeed lag behind the immediate and more visible changes in the biome. This lag occurs due to several factors that influence how quickly ecosystem processes can adjust to new plant communities introduced by invasive species. For example, changes in the C and N cycles in response to plant invasion often exhibit a time lag, whereby the ecosystem takes time to adjust to the shifts introduced by invasive species (Haubrock et al., 2022). This delayed response can stem from factors such as microbial adaptation and gradual litter accumulation, which modify nutrient cycling over time rather than immediately upon invasion. Moreover, during the early stages of decomposition, the allelochemicals released from invasive litter could inhibit the access of nearby plant species to essential nutrients, affecting nutrient cycling indirectly. As these chemicals break down over time, they can alter nutrient dynamics, favoring the invasive species while limiting the resources for native plants. When plant invasions occur, the immediate effects are often observed in biological variables such as the abundance and diversity of the plant and animal species within the biome. These biological changes are more rapidly apparent than ecosystem processes, which respond more slowly to new conditions (Hulme et al., 2013; Marchante et al., 2019). Our studies lasted only 6 months, which might be too short to detect significant changes in the life cycle of invasive plant litter. The effects of litter on nutrient cycling (e.g., nitrogen mineralization and nitrification) may only be detectable over a longer period. Secondly, these discrepancies can also be explained by the complexity of the underlying biological processes and their interactions (Čapek et al., 2018; Hättenschwiler and Vitousek, 2000). The nutrients in the *S. canadensis* and *P. arundinacea* litters were relatively abundant, promoting early enzymatic activities such as catalase in microbial activity. However, there was no significant change in urease activity associated with nitrogen cycling during litter decomposition, and the activity of invertase increased, but not significantly. The nutrients in the litter likely accelerated the growth of microorganisms, accelerating the decomposition of the litter. This interaction is a key component of nutrient cycling in ecosystems, such as nitrogen, phosphorus, and other minerals, providing excellent substrates for the growth of soil microorganisms. These nutrients are essential for the metabolic processes of bacteria, fungi, and other decomposers. As microorganisms access these nutrients, their populations can rapidly increase, enhancing their enzymatic activity and the overall rate of litter decomposition. Secondary metabolites, such as allelochemicals, phenolics, and other bioactive compounds produced by plants, can indeed slow down litter decomposition and nutrient cycling by inhibiting the soil enzyme activity and affecting the microbial communities (Ma et al., 2022; Scavo et al., 2018; Weidenhamer et al., 2023). Finally, differences in the soil type, soil depth, and the quantity and type of litter added also play a role (Bonanomi et al., 2016; Delgado-Baquerizo et al., 2015; Hobbie, 1992; Vahdat et al., 2011).

### 4.4 Effects of litter on soil feedback

Plant litter can have profound effects on the soil feedback mechanisms, influencing the soil properties, microbial communities, nutrient cycling, and plant growth (**Figure 5**). This feedback can be either positive or negative, depending on the quality and composition of the litter, as well as the specific ecological context (Feng et al., 2022; Herrera Paredes and Lebeis, 2016). The feedback between plants and soil is indeed a crucial driving force determining plant growth, physiology, and community composition (Bever et al., 1997; Mariotte et al., 2018; van der Putten et al., 2013). Our results exposed a vital outcome of soil conditioning on the biomass of native *P. arundinacea* and invasive *S. canadensis*. This is similar to the findings of many feedback studies that soil nutrients are accessible through the fast decomposition of nutrient-rich litter (from invasive species), increasing the plant nutrient availability in the next generation (Olson and Wallander, 2002). Nutrient-rich litter can enhance soil fertility by providing essential nutrients such as nitrogen, phosphorus, and potassium as it decomposes. Soil fertility can improve plant growth, produce more litter, and provide a positive feedback loop for nutrient enrichment.

In addition, positive litter-mediated feedback can favor the invasion of exotic plants, which often are more competitive for the nutrients released from decomposing plant litter. This process can lead to shifts in the plant community composition and further facilitate the spread of invasive species (Eppinga et al., 2011). Invasive species can create favorable conditions, such as an enhanced level of available nutrients in the occupied habitats, which can increase the probability of successfully invading another invasive species in the same habitat (Kuebbing et al., 2014; Mahla and Mlambo, 2019). In the context of exotic plants, an increasing number of studies specified that introducing non-native plants into new ecosystems can significantly affect the soil nitrogen and carbon cycling, which can positively or negatively affect the soil biota. These effects can either increase or decrease the rates of these essential processes, depending on various factors including the characteristics of the invasive species, the existing soil conditions, and the interactions with native species and soil microbes (Pellegrini et al., 2021; Qin et al., 2014; Zhang et al., 2019d). However, generalizing litter-mediated feedback through nutrients is complex due to the confounding effects of other chemical compounds and biotic interactions. These additional factors can lead to unexpected outcomes for future generations of plants (Hobbie, 2015). Most of the studies have demonstrated or suggested the potential for soil feedback to alter competition through soil nutrient and microbial community changes. In the present study, soil nutrients were also made available through the rapid decomposition of the nutrient-rich litter of *S. canadensis*. In contrast, the litter of *P. arundinacea* had a high concentration of structural carbohydrates, decomposed slower, and produced less positive plant–soil feedback (Ren et al., 2024; Wardle et al., 2004). Nutrient-mediated litter feedback is generally less specific to individual plant species than plant–soil feedback driven by specialized allelochemicals, resulting in the differential responses among plant species with varied growth strategies. Typically, fast-growing, nutrient-responsive species capitalize on nutrient-rich conditions, potentially shifting the composition of plant communities in their favor. Slow nutrient recycling, in contrast, can hinder plant species with more conservative resource-use strategies, as observed by Veen et al. (2019). Our findings similarly showed that, over time, the nutrient inputs from litter decomposition tended to favor the invasive *S. canadensis* over the native species. Moreover, distinct litter types may host unique litter microbiomes, with particular fungal communities developing that can create “home-field advantage” effects. In *S. canadensis*, the shifts in fungal populations associated with its litter have been shown to enhance the decomposition rates and nutrient availability (Lin et al., 2019). This process not only expedites nutrient cycling but can also establish microbial communities that reinforce the invasiveness of *S. canadensis* in ecosystems where it becomes established, amplifying its competitive edge against native flora.

## 5 Conclusions

The results of the study showed that soil environments such as mixed plots increase invasive plant litter decomposition. Significant variations in LMR were observed across different litter types—specifically invasive (*S. canadensis*), native (*P. arundinacea*), and their interactions. When decomposed individually, the *S. canadensis* litter exhibited a faster degradation rate compared with *P. arundinacea*. However, in mixed communities, the decomposition dynamics shifted, with *S. canadensis* experiencing a slower rate of decomposition while *P. arundinacea* decomposed more rapidly. This finding supports our hypothesis that, during the initial stages of litter decomposition, the rapid release of allelochemicals is a significant factor, whereas nutrient input becomes more prominent in the later stages of decomposition. Moreover, our results revealed that invaded soils negatively impacted the growth of *P. arundinacea*, with reductions of approximately 30% in plant height compared with native soils. This finding provides compelling evidence that litter can alter the soil ecosystem properties, promoting positive litter-mediated plant–soil feedback that favors the invasion of exotic species. The results of this research contribute to the understanding of the ecosystem-scale impacts of invasive plants and their direct and indirect effects, highlighting the interplay between native and invasive species and their associated decomposer communities.

In light of these findings, future research perspectives could:

- Investigate the long-term ecological impacts of invasive species on soil health, nutrient cycling, and plant community dynamics, which will provide deeper insights into the sustainability of ecosystems affected by invasions.
- Conduct comparative studies between different invasive and native species across various environmental contexts, which will help to elucidate the specific mechanisms through which invasives alter ecosystem processes.
- Examine the role of microbial communities in litter decomposition and nutrient cycling, which could reveal additional layers of complexity in plant–soil interactions, particularly in invaded *versus* native environments.

Addressing these future research perspectives could enhance our understanding of the ecological dynamics at play in invaded ecosystems and aid in the development of more effective management practices to combat the negative impacts of invasive plant species. This approach will be crucial for preserving biodiversity and maintaining the integrity of terrestrial ecosystems.

## Data Availability

The raw data supporting the conclusions of this article will be made available by the authors, without undue reservation.
